# The Disconnect between China's Public Health and Public Security Responses to Injection Drug Use, and the Consequences for Human Rights

**DOI:** 10.1371/journal.pmed.0050240

**Published:** 2008-12-09

**Authors:** Stephen Koester

## Abstract

Stephen Koester discusses a research article by Elizabeth Cohen and Joseph Amon that illustrates how injection drug users are caught between China's conflicting policies and practices toward drug use and HIV prevention and care.

## The Collision of Public Health and Public Security Responses

The HIV/AIDS epidemic has brought into sharp focus the contradictions between law enforcement and public health strategies aimed at socially marginalized populations such as drug users. Throughout the world, drug users are stigmatized as social deviants and often criminalized by the state. In addition to creating conditions that exacerbate bloodborne disease risk, this moralistic and legal approach runs counter to evidence-based and internationally accepted best practices for addressing substance use and HIV/AIDS. The result is that public health efforts are often compromised by law enforcement policies and practices that seek to limit illicit drug use by controlling and punishing drug users [1–3].

As illustrated in a paper by Elizabeth Cohen and Joseph Amon in this week's *PLoS Medicine* [4], China is a setting in which this contradiction in policy plays out. It is manifested by an increasingly pragmatic and responsive public health approach to HIV/AIDS that is occurring alongside a nationwide anti-drug campaign based on mandatory detoxification programs and re-education through labor centers (RELCs) [5,6]. On the one hand, recent studies suggest that the Chinese government is adopting progressive, evidence-based public health policies regarding HIV/AIDS prevention and care, as well as substitution therapy for drug use [5–7]. The central government has committed to funding 1,500 methadone maintenance therapy clinics and 140 needle and syringe programs by the end of 2008 [6], and results from pilot programs are promising [8–10].

On the other hand, however, the government is waging a “National People's War on Drugs” with the goal of increasing the number of people detained. According to Cohen and Amon, approximately 350,000 drug users were confined in 700 compulsory detoxification centers and 165 RELCs in 2005 [4]. Officially, sentences in these facilities range from three to six months in the detoxification centers and two to three years in RELCs. In practice, however, sentences are often of indeterminate length [4,11]. In 2007 the Chinese government passed an anti-drug law that is to replace RELCs with a new form of detention combining one- to two-year sentences in compulsory detoxification centers with up to three years of rehabilitation in the community [4]. This “less publicized aspect of the country's war on drugs has [resulted in] the lost opportunities of those mired in a cycle of arrest, detoxification, release, relapse and arrest. Many drug users live their lives in constant fear of police raids, endure difficult conditions and long periods away from friends and family while in confinement and suffer high rates of unemployment and discrimination when they are not in government facilities” [11].

**Linked Research Article**

This Perspective discusses the following new study published in *PLoS Medicine*:

Cohen JE, Amon JJ (2008) Health and human rights concerns of drug users in detention in Guangxi Province, China. PLoS Med 5(12): e234. doi:10.1371/journal.pmed.0050234

Based on their review of Chinese government legislation and policy documents, and using interviews with recently detained injection drug users and officials in Guangxi Province, Elizabeth Cohen and Joseph Amon find evidence of antinarcotics policies and practices that may compromise the health and human rights of drug users.

The article by Cohen and Amon [4] highlights some of the consequences of this less publicized public security–led anti-drug strategy, concluding that it routinely violates the human rights of drug users. Their brief assessment is based on a legal and policy review of Chinese government anti-narcotics legislation and other pertinent policy documents, as well as semi-structured interviews with key informants and injection drug users (IDUs) in two cities in China's Guangxi province. Guangxi borders Vietnam and is one of China's two main ports of entry for heroin from the Golden Triangle, the area of Myanmar, Thailand, and Laos that is second only to Afghanistan in heroin production. The province has a large number of drug users and one of the country's highest rates of HIV prevalence [6]. Key informants included health professionals from various levels of government, representatives of domestic and foreign nongovernmental organizations, a physician working in a detoxification center, and a former RELC guard. The authors interviewed 15 male and four female IDUs who had been released from detox centers or RELCs within the past 18 months. Twelve IDUs self-reported being HIV positive; seven were unaware of their status.

Interviews with IDUs revealed experiences of everyday violence that resulted from national anti-drug policies carried out by local authorities. IDUs described living in constant fear of arrest or being reported by their neighbors. IDUs believed that the consequences of arrest were uncertain and dependent on whether local authorities had made their arrest quotas or whether detox centers and RELCs had room for additional occupants. This fear, they said, dissuaded them from accessing HIV prevention programs, sterile syringes from pharmacies, and methadone maintenance therapy.

According to Cohen and Amon, local authorities determined an IDU's assignment to a mandatory detox program or RELC. There was no due process and no opportunity to appeal a decision. Living conditions were often dire. IDUs complained that the centers repeatedly tested them for HIV but did not provide results. Key informants confirmed this, and the former guard explained that guards used female detainees' test results to determine whether to use a condom when having sex with them.

IDUs reported that health care in detention centers was very limited; there was no monitoring of HIV-positive detainees' status and access to antiretroviral therapy was uncertain. IDUs reported that unsafe sex and injection drug use occurred in the centers.

## Implications

The assessment by Cohen and Amon is not without flaws. Its contribution is compromised by the brief period of fieldwork and the opportunistic approach to recruiting participants. Additionally, interviews with 19 IDUs, some lasting as long as three hours, suggest that there is much more to this story than reported here. More troubling, however, is that this assessment was not reviewed and approved by an external human research participants review board. The authors relied solely upon an internal review of their research plan and measures taken to protect study participants. As noted in the research article, the advisory committee of *PLoS Medicine* concluded that this review was hardly ideal. Nongovernmental organizations conducting research on topics as sensitive as the plight of IDUs should adhere to the same standards for ensuring the ethical conduct of research and protection of human study participants as other researchers.

Nevertheless, this study is useful because it gives a voice, however limited, to drug users caught within China's conflicting policies and practices toward drug use and HIV prevention and care. It demonstrates the potential of qualitative research, even under less than opportune circumstances, to uncover the meaning and consequences of top-down policies on people's lives. Although the study did not include observations inside mandatory detox centers or RELCs, or interviews with drug users currently incarcerated, interviews with users who had past experiences with these facilities provide a compelling window into the everyday experience of drug users enmeshed in this system. Their accounts provide a sobering reality check to reports heralding China's “bold steps to scale up” HIV prevention and treatment [7]. They remind us that seemingly progressive public health policies cannot fully be effective when carried out within a context of punishment and fear.

## Looking Forward

China's contradictory response to drug use is not unique. Throughout the world—in Western democracies as well as in more authoritarian governed nations—researchers and health practitioners are working to limit the damage of drug use and control the suffering caused by HIV/AIDS. And in many cases, they are operating within a state-sponsored system that criminalizes and intimidates the very people they attempt to help. Listening to the powerless, marginalized recipients of these conflicting policies exposes this contradiction and provides a powerful argument for change. Carefully constructed qualitative studies can be useful tools for detailing and monitoring the tragic and unnecessary consequences of law enforcement responses to drug use.

Fortunately, there are indications that China's policies toward drug users are evolving and that there is increasing dialogue and cooperation between public health and public security authorities at all levels of government. This is encouraging, and will hopefully lead to more humane and thus more effective ways to reduce the harm caused by drug dependence.

## Abbreviations

IDU: injection drug user
RELC: re-education through labor center
